# Using Remote Fields for Complex Tissue Engineering

**DOI:** 10.1016/j.tibtech.2019.07.005

**Published:** 2019-08-19

**Authors:** James P.K. Armstrong, Molly M. Stevens

**Affiliations:** 1 Department of Materials, Department of Bioengineering, and Institute for Biomedical Engineering, Imperial College London, London, SW7 2AZ, UK

## Abstract

Great strides have been taken towards the *in vitro* engineering of clinically relevant tissue constructs using the classic triad of cells, materials, and biochemical factors. In this perspective, we highlight ways in which these elements can be manipulated or stimulated using a fourth component: the application of remote fields. This arena has gained great momentum over the last few years, with a recent surge of interest in using magnetic, optical, and acoustic fields to guide the organization of cells, materials, and biochemical factors. We summarize recent developments and trends in this arena and then lay out a series of challenges that we believe, if met, could enable the widespread adoption of remote fields in mainstream tissue engineering.

## The Current Status of Tissue Engineering: Clinical Solutions but Little Structure

Tissue engineers today can draw on a host of new scientific and technological tools, such as cell reprogramming, gene editing, and advanced cell screening [1]. These advances have enabled unprecedented levels of biological control and characterization, however, it is striking to note how the core principles of this discipline have remained largely unchanged from the early tissue engineering experiments carried out by Bell and coworkers in 1979 [2]. In particular, cells, biomaterials, and biochemical factors are still widely held up as the three pillars of tissue engineering. If we set aside certain practical flaws, such as nutrient transport gradients [3], then we can consider most tissue engineering protocols to be equilibrium systems: cells dispersed homogenously throughout isotropic materials, with nutrients and biochemical factors supplied in a global fashion by bulk diffusion from the surrounding culture medium. The absence of any imposed constraints or directionality means that these strategies generally yield homogenous tissue constructs that exhibit very little structural organization.

Evidently, this situation is far removed from the vivid complexity of natural systems. Tissues often exhibit multiscale and hierarchical structure, with anisotropic, inhomogeneous, and directional arrangement of both cells and extracellular matrix components. Structural organization is almost always inextricably linked to physiological function, for instance, the anisotropic packing of myofibers ensures directional actuation of skeletal muscle [4], the orientation of cardiomyocytes guides the propagation of contractile waves in myocardial tissue [5], and the zonal organization of cartilage and bone allows effective load transmission across the osteochondral interface [6]. Moreover, it is widely appreciated that tissue grafts engineered with mature vasculature and structural profiles that match the defect site have an improved chance of successful integration and survival [7,8]. It is clear that the functional performance of an engineered tissue graft will be negatively impacted by a failure to address structural complexity. Indeed, it is our view that *in vitro* tissue engineering is unlikely to become established as a mainstream clinical practice without addressing the issue of structural organization.

This point can be illustrated by considering the nature of some of the major tissue engineering products and procedures that have reached the clinic: skin grafts (e.g., Epicel®, Apligraf®, Dermagraft®), cartilage implants (e.g., MACI®), and corneal sheets (e.g., Holoclar®) [1]. The clinical success of these products is owed to the fact that they can still perform certain functions without possessing optimal tissue structure. Skin grafts can provide a basic physical barrier for patients with burns or ulcers, despite lacking natural features, such as sweat glands. Cellularized implants that fill small articular cartilage defects can improve joint function and relieve pain, despite these constructs lacking the intricate organization of cells and extracellular matrix fibers. Meanwhile, corneal sheets can be used to improve visual acuity in cases of limbal stem cell deficiency, despite the absence of zonal organization. These examples serve as a marker of the current status of tissue engineering: products that offer clinical benefits ‘despite’ a lack of structural organization.

## Next-Generation Tissue Engineering: Building Structural Complexity

Whether we are looking to improve the clinical utility of existing products or seeking translational solutions for more structurally demanding targets (e.g., liver, neural, cardiac tissue), it is critical that the next generation of tissue engineers focus on methods that can fully replicate physiological functions by faithfully recreating native structural complexity. There are a host of fabrication tools that can be used to spatially arrange cells, materials, and biochemical factors. For instance, 3D bioprinting is commonly used to create precision architecture and patterned multicellular structures [9]. Techniques such as aligned electrospinning [10], melt electrospinning writing [11], and unidirectional freeze drying [12] can be used to create anisotropic substrates, while materials can also be molded [13], layered into zonal structures [14], or cast with mechanical, compositional, or morphogen gradients [15]. These fabrication-driven approaches are either used to spatially organize components prior to tissue culture or as a means of creating material or biochemical cues that can drive directional or local cell responses during tissue culture.

**Figure 1:**
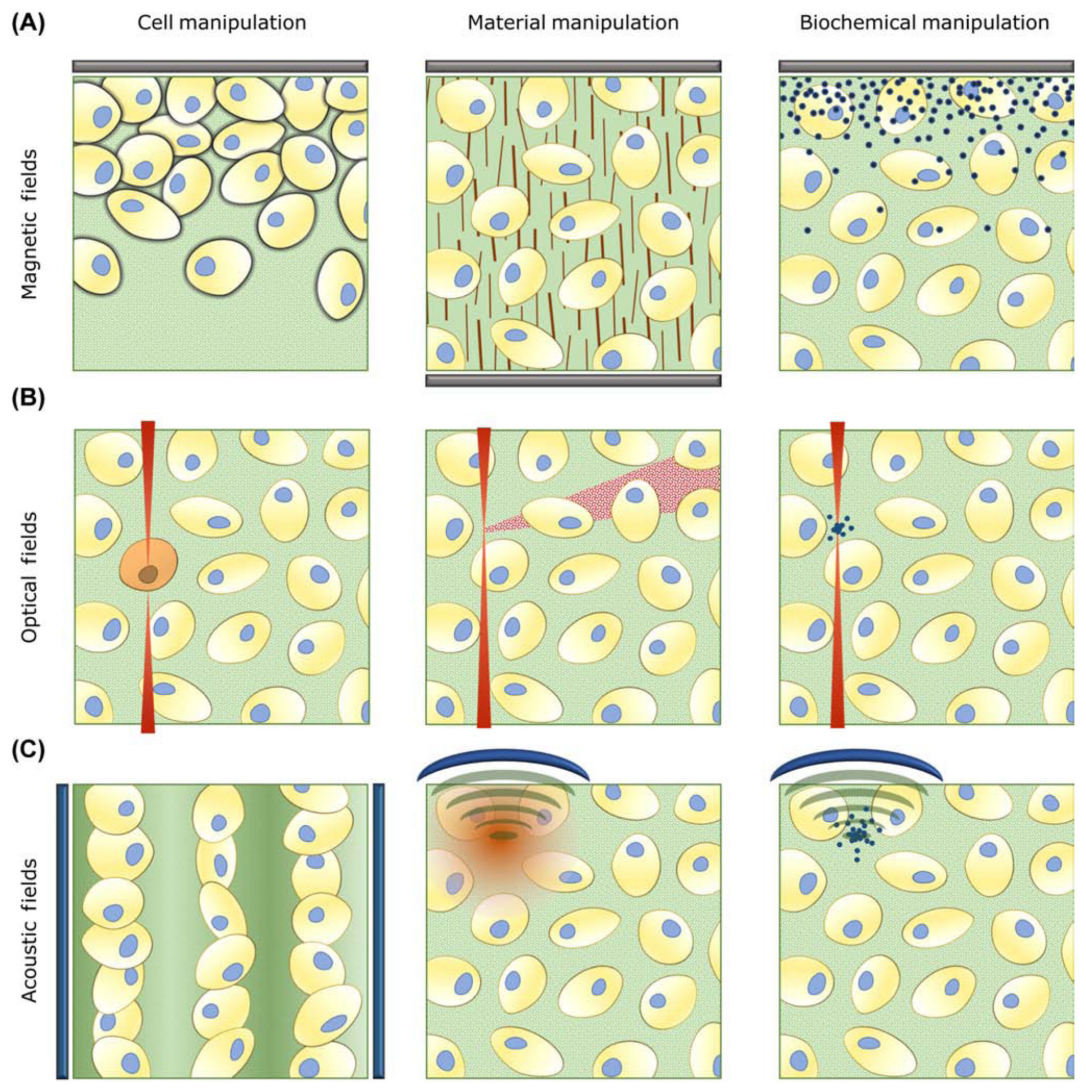
Key Figure: Remote Fields in Tissue Engineering: Literature Examples and Inspiration. (A) Magnets (shown in gray) offer a relatively simple and accessible method for manipulating the position of magnetized cells [28], aligning matrix fibers [17], or patterning growth factor gradients [22]. (B) Focused optical fields (shown in red) provide high spatial resolution that can be used for optogenetic modulation of cells [40], photodegradation of materials [34], or local release of biochemical factors [35]. (C) Acoustic fields can be generated by piezotransducers (shown in blue) in order to pattern bulk cell populations [45], modulate fiber microstructure [57], or locally release biochemical factors [59]. Many of these approaches, along with related technologies, are already used to engineer complex tissue structures, while others require further development, optimization, or scale up.

An alternative strategy that has been investigated for complex tissue engineering is the use of externally applied forces. For example, electrical or mechanical stimulation can be used to guide cell alignment or differentiation during tissue engineering [16]. These examples use apparatus that directly interface with either the cells, media, or tissue constructs. In this article, we wish to highlight a related strategy that has recently come to prominence: the use of remotely applied force fields (see Glossary) (e.g., optical, acoustic, magnetic) that can be used to guide structural complexity without any tangible contact. While appreciating that these fields can be used to promote bulk effects (e.g., global cell differentiation), we have focused on strategies that seek to disrupt the equilibrium balance of cells, materials, or biochemical factors (Figure 1, Key Figure). In particular, we highlight recent approaches that use remote fields to: (i) spatially assemble different tissue engineering components; (ii) initiate local responses, such as cell differentiation or material degradation; or (iii) exert directional responses, such as cell or matrix fiber alignment.

### Magnetic Fields

**Figure 2:**
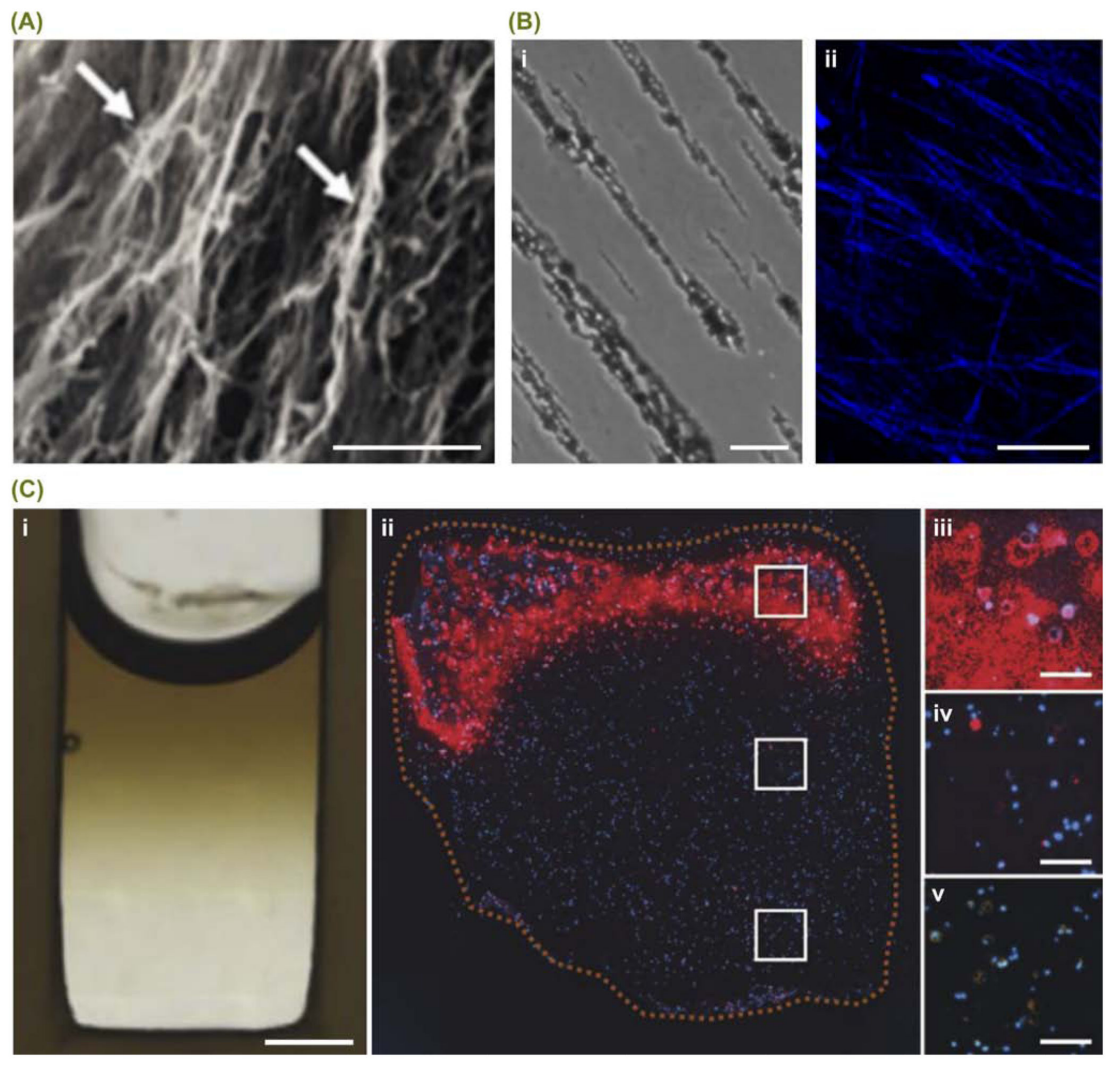
Magnetic Fields for Manipulating Biomolecules. Magnetic fields are particularly well suited to the redistribution of nanoscale entities, such as biomolecule aggregates or magnetically susceptible nanoparticles. (A) For example, Eguchi and coworkers recently used an 8 T magnetic field to orient collagen fibers (white arrows) during self-assembly and gelation. Scale bar = 50 μm. The aligned structures were capable of guiding Schwann cell orientation and promoting nerve regeneration in both *in vitro* and *in vivo* models. Reproduced, with permission, from [17]. (B) The addition of superparamagnetic nanoparticles to the hydrogel precursor can allow fiber alignment with much weaker fields. For example, a recent report showed that a 26 mT magnetic field could be used to form hydrogels containing (i) aligned superparamagnetic nanoparticle strings, and (ii) oriented collagen fibers (blue). Scale bars = 50 μm. Reprinted, with permission, from M. Antman-Passig and O. Shefi *et al*. Nano Letters Copyright 2016 American Chemical Society [18]. (C) Superparamagnetic nanoparticles can also be used as field-responsive carriers for biomolecular cargo. (i) For instance, Li and coworkers used a 700 mT magnetic field to form an agarose hydrogel bearing a gradient of superparamagnetic nanoparticles (dark brown). Scale bar = 2 mm. By preloading the nanoparticles with bone morphogenetic protein 2 and co-encapsulating human mesenchymal stem cells into the agarose hydrogel, the gradient was used to engineer osteochondral tissue constructs. Immunostaining for osteopontin (red), counterstained with DAPI (blue) across (ii) the whole construct, (iii) the bone end, (iv) the middle section, and (v) the cartilage end. Scale bars = 200 μm. Reproduced, with permission, from [22].

Strong magnetic fields have been widely used to orient matrix fibers during gelation, for example, Eguchi and coworkers recently used an 8 T field to fabricate aligned collagen hydrogels that could guide Schwann cell orientation [17] (Figure 2A). However, the recent trend has seen the introduction of magnetically susceptible components that allow alignment using much weaker magnetic fields. For instance, Antman-Passig and coworkers used superparamagnetic nanoparticles to guide the alignment of collagen fibers in a 26 mT field [18] (Figure 2B). This aligned system was used for neural cell orientation, while a similar approach was employed by Betsch and coworkers for the 4D bioprinting of cartilage tissue [19]. Meanwhile, magnetic surfactant conjugation has been used to magnetize proteins [20], and superparamagnetic nanoparticles have been used as field-responsive carriers for RNA and proteins [21–23]. The latter approach was used by Li and coworkers to create gradients of bone morphogenetic protein 2 (BMP-2) within cellularized hydrogels, in order to promote localized osteogenesis and mineralization during osteochondral tissue engineering [22] (Figure 2C).

An interesting route to cellular manipulation was recently reported by Tasoglu and coworkers, who used external magnetic fields for the levitational self-assembly of cell-seeded materials in a paramagnetic suspension [24]. A more common approach is direct cell magnetization, however, this strategy necessitates the use of cytocompatible protocols that can label cells with a large quantity of paramagnetic material [25]. Bulk cell magnetization has been used to engineer vocal folds [26], secretory epithelial organoids [27], and embryoid bodies [28], however, superparamagnetic nanoparticles can also interact with cells in a more refined manner. Historically, superparamagnetic nanoparticles have been targeted to specific integrin receptors or ion channels on the cell membrane surface allowing remote field actuation [29]. More recently, magnetic fields have been used to manipulate the position of functionalized, intracellular superparamagnetic nanoparticles in order to modulate processes such as cytoskeletal assembly, mitochondrial dynamics and gene expression [30–32]. With further development, this method could potentially offer a remotely activated magnetogenetic switch for complex tissue engineering.

### Optical Fields

**Figure 3:**
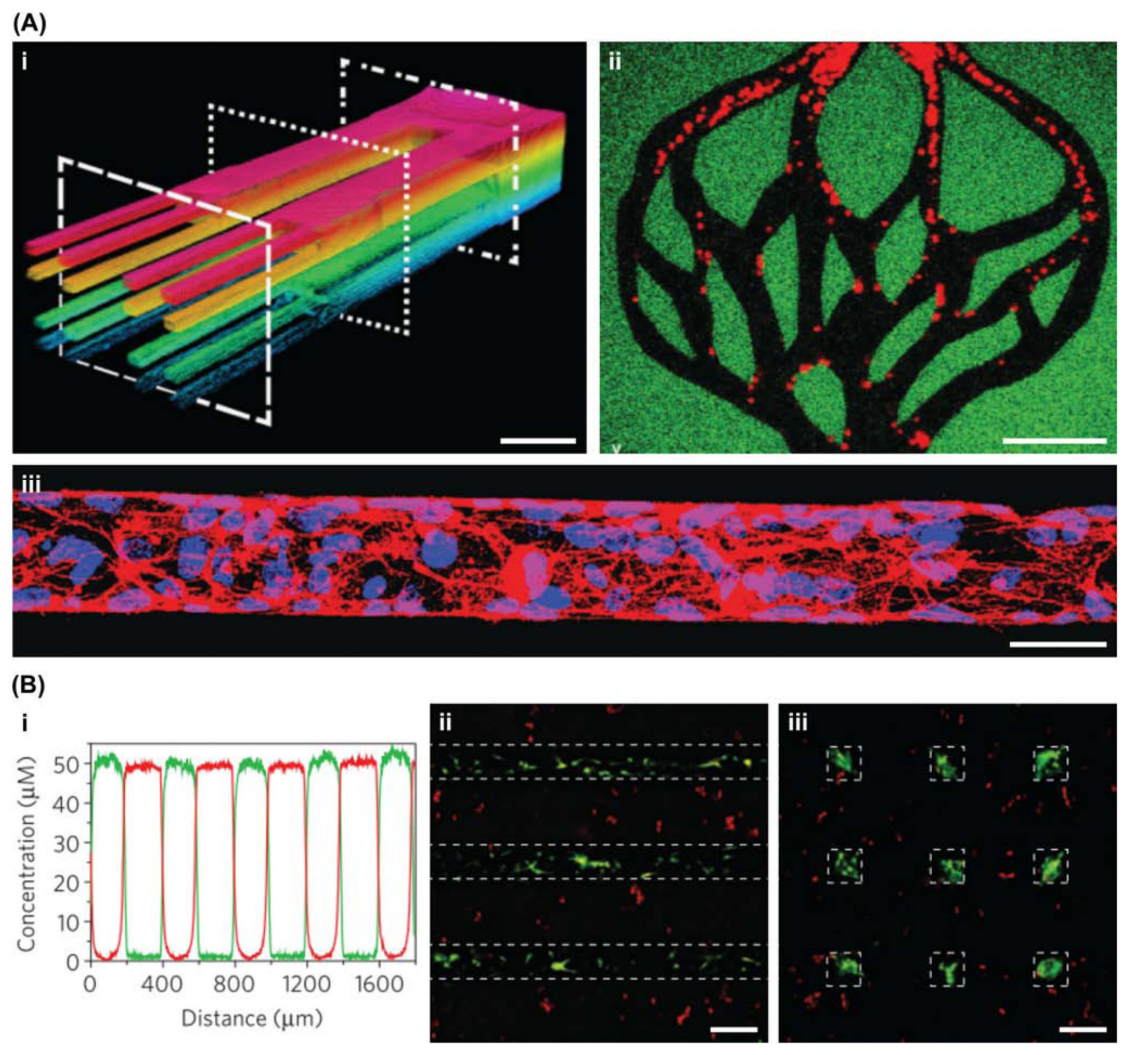
Optical Fields for Manipulating Materials. Light can be used for the formation or cleavage of bonds, a property that makes it highly suited to the patterning of material systems. (A) For example, Arakawa and coworkers used a light-mediated subtractive manufacturing technique based on molecular photolysis to create complex 3D networks of perfusable microvessels. The vessels could be perfused with fluorescent microbeads, shown with (i) z-depth-dependent color coding and (ii) as distinct particles (red). Scale bars = 100 μm. (iii) The channels were seeded with endothelial cells to form vascular networks, shown here stained with phalloidin (red) and Hoechst 33342 (blue). Scale bar = 50 μm. Reproduced, with permission, from [34]. (B) DeForest and Tirrell used two different photochemistries: one wavelength to reversibly anchor proteins to the surrounding hydrogel and a second wavelength to remove the immobilized protein. (i) This method was used to sequentially pattern two different proteins (red and green traces) with high precision and minimal overlap. (ii) Immunostaining for osteocalcin (green) counterstained with CellTracker (red) showed osteogenesis of mesenchymal stem cells only in regions of vitronectin patterning (dashed lines), (iii) which could be reversed by selectively removing protein to leave smaller regions of vitronectin (dashed squares). Scale bars = 200 μm. Reproduced, with permission, from [35].

Tissue engineering components can be manipulated with much higher precision using optical technologies. Light irradiation can be used to form, cleave, or rearrange chemical bonds, a characteristic ideally suited to patterning materials [33]. Historically, simple photomask-based systems have been used to present an uneven distribution of light, however, the recent trend has been towards more dynamic 3D patterning techniques, such as multiphoton lithography. This method was used by Arakawa and coworkers to precisely sculpt vascular networks in hydrogels crosslinked with photodegradable peptides [34] (Figure 3A). A similar method was used by DeForest and Tirrell, who used two bio-orthogonal photochemistries to immobilize and release proteins within a synthetic hydrogel (Figure 3B). Applying this approach to vitronectin enabled the reversible and spatiotemporal differentiation of human mesenchymal stem cells [35]. An alternative method is the use of localized heating caused by infrared irradiation to trigger temperature-mediated processes. This strategy was employed by Stowers and coworkers to release liposomal cargo that could either stiffen or soften alginate hydrogels with spatiotemporal control [36].

Local heating by infrared light was also used by Martin-Saavedra and coworkers to spatiotemporally trigger the transgene expression of vascular endothelial growth factor (VEGF) [37]. This study used a heat-activated gene switch, however, a more direct method for optically controlling cells is the use of optogenetic technology. Indeed, optogenetics has enabled the direct photoactivation of processes such as neurite outgrowth, myogenic differentiation, and angiogenesis [38,39], while Reis and coworkers recently reported that optoepigenetic probes could be used for light-controlled gene expression [40]. While optogenetic/optoepigenetic tissue engineering requires further development, these reports offer the enticing prospect of using optical switches to guide complex tissue formation. Meanwhile, other light-based technologies have been used to precisely assembly different tissue engineering components. For instance, optical tweezers can trap and maneuver individual cells or microtubules into customized arrays [41,42]. Although optical tweezers offer extremely high spatial precision, it is clear that significant improvements in throughput are required if this method is to be applied to the engineering of full-size tissue constructs.

### Acoustic Fields

**Figure 4:**
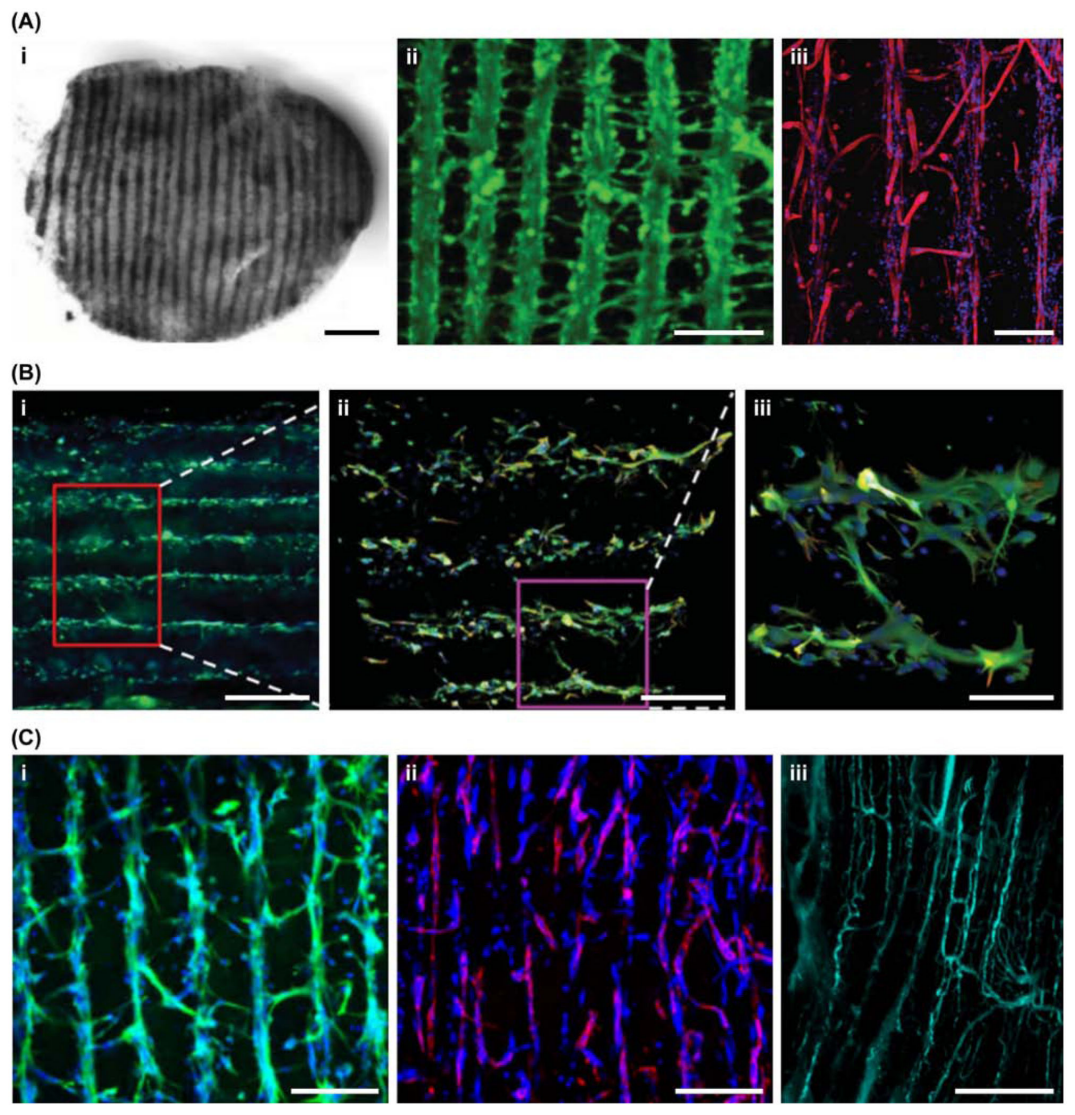
Acoustic Fields for Manipulating Cells. Ultrasound standing waves are particularly well suited for creating geometric assemblies of microscale entities, which makes them highly applicable to remote cell patterning. (A) For example, Armstrong and coworkers used this approach to pattern myoblasts throughout collagen-based hydrogels for the tissue engineering of aligned skeletal muscle. (i) Low magnification bright field image of a patterned muscle tissue, scale bar = 500 μm. (ii) Confocal fluorescence microscopy image of calcein-stained myoblasts (green), scale bar = 200 μm. (iii) Confocal fluorescence microscopy image of myotubes immunostained for skeletal myosin (red) and counterstained with DAPI (blue), scale bar = 300 μm. Reproduced, with permission, from [45]. (B) Bouyer and coworkers used acoustic levitation to form layers of neuroprogenitors in fibrin hydrogels for neural tissue engineering. Immunostaining for Tuj 1 (green) and nestin (red), counterstained with DAPI (blue), (i) scale bar = 500 μm, (ii) scale bar = 250 μm, (iii) scale bar = 100 μm. Reproduced, with permission, from [48]. (C) Kang and coworkers used two orthogonal standing waves to create 3D arrays of collateral cylindroids containing both endothelial cells and adipose stem cells. (i) Immunostaining for vascular endothelial cadherin (green) counterstained with DAPI (blue), scale bar = 200 μm. (ii) Immunostaining for α smooth muscle actin (red) counterstained with DAPI (blue), scale bar = 200 μm. (iii) Image of aligned vessels, perfused with fluorescent dextran (green), 1 week after subcutaneous transplantation, scale bar = 200 μm. Reproduced, with permission, from [50].

A more high-throughput method for cell manipulation is acoustic patterning. This method typically uses one or more ultrasound standing waves to create uneven pressure fields that can pattern cells *en masse* into well-defined geometric assemblies [43]. Early work in this field focused on patterning cells on 2D culture substrates, however, a major development in this area was the use of hydrogelation to encapsulate the patterned cell arrays [44]. This method allowed cell arrays to be preserved for long-term *in vitro* tissue engineering after removal of the acoustic field. Since 2016, devices have been developed to generate lines of myoblasts for skeletal muscle tissue engineering [45] (Figure 4A), assemblies of beating cardiomyocytes for cardiac tissue engineering [46,47], levitated sheets of neuroprogenitors for neural tissue engineering [48] (Figure 4B) and arrays of endothelial cells for neovascularization [49,50] (Figure 4C). These examples use simple geometric arrays to create lines, columns, or sheets, however, in the future it may be possible to create customized tissue architecture by employing more flexible holographic assembly routes [51,52].

A limitation to acoustic cell patterning is that the forces generated are relatively weak [53]. As a result, acoustic patterning can be readily disrupted by factors such as mechanical agitation, thermal currents, acoustic streaming, viscosity, and gravity [54]. Moreover, the forces used for patterning are proportional to the object volume and thus it can be extremely challenging to pattern nanoscale entities, such as biochemical factors or matrix fibers. This limitation has led to the development of strategies that use microscale carriers to host biomolecular cargo or template material fabrication [55,56]. An alternative approach is the use of focused ultrasound that can provide highly localized stimuli capable of modulating different tissue engineering components. The local heating caused by focused ultrasound has been used to modulate fibril size during collagen gelation [57] and trigger the transgene expression of growth factors (BMP-2 and VEGF) in a spatially controlled manner [58]. Focused ultrasound can also be used to initiate non-thermal effects, such as the liberation of growth factors from acoustically responsive droplets or scaffolds [59,60].

## Concluding Remarks and Future Perspectives

It is evident that remote field systems have the potential to greatly advance *in vitro* tissue engineering. However, the convergence of magnetic, optical, and acoustic technologies with *in vitro* tissue engineering is a relatively new development and, as a result, many studies exist only in the academic sphere and are far from disrupting the clinical *status quo*. Here, we propose three key challenges that must be addressed in order to realize the benefit of remote fields in translational tissue engineering (see Outstanding Questions). First, it is imperative that ‘proof-of-concept’ studies using arbitrary feature dimensions and model cell types are replaced with methods that enable the engineering of mature, multicellular tissues with native structural dimensions. For instance, while ultrasound standing waves have been used to create multilayer sheets of endothelial cells in collagen hydrogels [44], the next step for this technology is the creation of functional networks of blood vessels, replete with support cells (e.g., smooth muscle cells), formed not in a bare hydrogel but in a real tissue structure (e.g., muscle, bone, liver).

The second challenge is to use materials and protocols that support remote manipulation without affecting other aspects of the engineered tissue. This statement is made in light of the fact that many remote field technologies necessitate the use materials with defined properties: certain levels of optical transparency, viscosity, magnetic susceptibility, photoresponsivity, or acoustic attenuation. As a result, many strategies rely on either: (i) a customized material, designed and synthesized with the appropriate set of characteristics; or (ii) the selection of an existing material system that has compatible properties within an operational parameter space. While a carefully designed or selected material can evidently be used to enable remote organization of structural features, this benefit must not be at the expense of the biological, physical, and mechanical properties required to support cell survival, differentiation, and extracellular matrix production. Ideally, a tissue engineering protocol that is recognized as the academic or clinical gold standard would be used with remote field application as the sole change to the established procedure.

The final challenge is to improve the accessibility of remote field instrumentation. Apparatus is often assembled in house [45,55], however, the need for users to assemble and operate their own devices restricts usage to a small number of groups with specialist expertise. This situation could be alleviated by more active dissemination of academic knowledge through protocols and methods papers, or by making devices available through user collaboration or product commercialization. Alternatively, many remote field technologies use high-end equipment that is already commercially available, such as multiphoton lithography [35], optical tweezers [41], or focused ultrasound systems [60]. These systems, which can require considerable expense and expertise to operate and maintain, tend to be sold as multifunctional apparatus rather than tailored to particular end-user applications. Therefore, a major challenge can be tuning and integrating commercial apparatus to meet biological requirements (cytocompatibility, sterility, *etc.*). Overall, the creation of more integrated, accessible technologies would enable research groups around the world to embrace remote fields as a mainstream tool for complex tissue engineering.

## Highlights

Remote fields have recently emerged as an alternative to contact force application and microfabrication in the engineering of complex tissue structures.

New methods have been developed that use magnetic fields to manipulate nanoparticles for biochemical patterning, material assembly, and biological actuation.

A series of recent reports in optical manipulation have shown high-level control over gene expression, epigenetics, material degradation, and reversible protein immobilization.

Recent advances have been made in preserving acoustically patterned cell arrays, which have enabled the engineering skeletal muscle, cardiac, and neural tissue.

## Glossary

**Acoustic attenuation:** the loss of energy of a sound wave as it propagates through a medium.

**Acoustic patterning:** the use of sound waves to create pressure gradients that can move particles (or cells) into nodes or antinodes.

**Focused ultrasound:** a technique that uses lenses or curved transducers to create local regions of high intensity sound energy with frequencies greater than 20 kHz.

**Force fields:** vector maps describing the noncontact forces experienced by particles at different spatial coordinates.

**Multiphoton lithography:** a technique that uses a focused laser to drive local changes (e.g., photocrosslinking, photopolymerization, photodegradation) with electronic excitation requiring the near-simultaneous absorption of two or more photons.

**Optical tweezers:** a technique that uses highly focused laser beams to spatially trap and manipulate small particles with a refractive index that is different to the surrounding medium.

**Optoepigenetic:** the use of light to control epigenetic mechanisms, for instance, by using photochromic inhibitors of chromatin-modifying enzymes.

**Optogenetic:** the use of light to modulate molecular events in living cells using genes encoding photoresponsive proteins.

**Paramagnetic:** a class of magnetism in which a material has unpaired electrons that can align with an external magnetic field.

**Superparamagnetic:** a magnetic behavior exhibited by small ferrimagnetic/ferromagnetic materials, which have single magnetic domains that can align with an external magnetic field.

**Transgene expression:** the biosynthesis of functional products (usually proteins) from genes artificially introduced into a cell.

**Ultrasound standing wave:** a sound wave that oscillates with a frequency of more than 20 kHz but has stationary pressure nodes.

## Outstanding Questions

Can we progress from ‘proof-of-concept’ studies towards the engineering of clinically relevant tissues with native multicellularity and natural feature dimensions?

Can we avoid a reliance on customized systems and instead develop methods that employ materials capable of supporting both structural organization and tissue development?

Can we move away from specialized techniques towards more accessible technologies, in order to allow adoption of remote fields in mainstream tissue engineering?
